# Sinc-Chebyshev Collocation Method for a Class of Fractional Diffusion-Wave Equations

**DOI:** 10.1155/2014/143983

**Published:** 2014-04-01

**Authors:** Zhi Mao, Aiguo Xiao, Zuguo Yu, Long Shi

**Affiliations:** ^1^Hunan Key Laboratory for Computation and Simulation in Science and Engineering and Key Laboratory of Intelligent Computing and Information Processing of Ministry of Education, Xiangtan University, Xiangtan, Hunan 411105, China; ^2^Mathematics and Information Engineering Department, Tongren University, Tongren, Guizhou 554300, China

## Abstract

This paper is devoted to investigating the numerical solution for a class of fractional diffusion-wave equations with a variable coefficient where the fractional derivatives are described in the Caputo sense. The approach is based on the collocation technique where the shifted Chebyshev polynomials in time and the sinc functions in space are utilized, respectively. The problem is reduced to the solution of a system of linear algebraic equations. Through the numerical example, the procedure is tested and the efficiency of the proposed method is confirmed.

## 1. Introduction

Fractional models have been increasingly shown by many scientists to describe adequately the problems with memory and nonlocal properties in fluid mechanics, viscoelasticity, physics, biology, chemistry, finance, and other areas of applications [1–6]. In particular, the fractional diffusion-wave equation has been used to model many important physical phenomena ranging from amorphous, colloid, glassy, and porous materials through fractals, percolation clusters, and random and disordered media to comb structures, dielectrics and semiconductors, polymers, and biological systems [7–10]. It is a generalization of the classical diffusion-wave equation by replacing the integer-order time derivative with a fractional derivative of order *α* (1 < *α* < 2). This equation can be derived from the anomalous superdiffusion in continuous time random walk which is generally non-Markovian processes [11].

Although the considerable work on the numerical solution of fractional diffusion equations has been done [12–15], there are very limited numerical methods for solving the fractional diffusion-wave equations [16–18]. However, all the above mentioned papers dealt with the fractional diffusion-wave equations by finite difference methods. It is well known that any algorithm based on the finite difference discretization of a fractional derivative has to take into account its memory or nonlocal structure; thus this means a high storage requirement [19].

In the present paper, we consider the following differential equation with the Caputo fractional derivative and a variable coefficient:

(1) ∂αu(x,t)∂tα=a(x,t)∂2u(x,t)∂x2+f(x,t),a<x<b,  0<t≤τ,

with the initial conditions,

(2) $$\begin{array}{c} u\left(x,0\right)=\phi \left(x\right),\;\;\frac{\partial u\left(x,0\right)}{\partial t}=\psi \left(x\right),\;a<x<b, \end{array}$$

and the boundary conditions,

(3) $$\begin{array}{c} u\left(a,t\right)=0,\;u\left(b,t\right)=0,\;0<t\leq \tau , \end{array}$$

where *x* ∈ [*a*, *b*] and *t* ∈ (0, *τ*] are space and time variables, respectively, *a*(*x*, *t*) is a continuous function, and *f*(*x*, *t*) denotes the field variable. For 1 < *α* < 2, the fractional equation (1) is known as the fractional diffusion-wave equation which fills the gaps between the diffusion equation and wave equation [16, 20].

We develop a sinc-Chebyshev collocation method to solve numerically problem (1) with (2) and (3). Since a fractional derivative is a nonlocal operator, it is natural to consider a global scheme such as the collocation method for its numerical solution [19, 21]. The required approximate solution is expanded as a series with the elements of shifted Chebyshev polynomials in time and sinc functions in space with unknown coefficients. By utilizing the collocation technique and some properties of the shifted Chebyshev polynomials and sinc functions, the problem is reduced to the solution to a system of linear algebraic equations. And a matrix representation of the system is obtained to calculate the solution. The presented method is effective and convenient.

The remainder of this paper is organized as follows: in the next section, we introduce some necessary definitions and relevant results for developing this method.  Section 3 is devoted to constructing and analyzing the numerical algorithm. As a result, a system of linear algebraic equations is formed and the solution of the considered problem is obtained. In Section 4, the numerical example is given to demonstrate the effectiveness and convergence of the proposed method. A brief conclusion is given in the final section.

## 2. Notations and Some Preliminary Results

In this section, we introduce some basic definitions and derive several preliminary results for developing the presented method.

### 2.1. The Caputo Fractional Derivative

**Definition 1 (see [22])**

Let *α* ∈ ℝ_+_. The operator *J*_*a*_^*α*^ defined on *L*_1_[*a*, *b*] by

(4) $$\begin{array}{c} J_{a}^{\alpha }f\left(t\right)=\frac{1}{\Gamma \left(\alpha \right)}\overset{t}{\underset{a}{\int }}{\left(t-s\right)}^{\alpha -1}f\left(s\right)ds‍ \end{array}$$

for *a* ≤ *t* ≤ *b* is called the Riemann-Liouville fractional integral operator of order *α*. For, *α* = 0, we set *J*_*a*_^0^ : = *I*, that is, the identity operator.

**Definition 2 (see [22])**

Let *α* ∈ ℝ_+_ and *n* = ⌈*α*⌉. The Caputo fractional differential operator ^*C*^ *D*_*a*_^*α*^ for *a* ≤ *t* ≤ *b* is defined as

(5) DaαCf(t)=Jan−αDnf(t)=1Γ(n−α)∫at(t−s)n−α−1f(n)(s)ds‍.

### 2.2. The Composite Translated Sinc Functions

The sinc functions and their properties are discussed in [23, 24]. For any *h* > 0, the translated sinc functions with equidistant space nodes are given as

(6) $$\begin{array}{c} S\left(k,h\right)\left(z\right)=\text{sinc}\left(\frac{z-kh}{h}\right),\;k=0,\pm 1,\pm 2,\ldots , \end{array}$$

where the sinc functions are defined on the whole real line by

(7) $$\begin{array}{c} \text{sinc}\left(x\right)=\{\begin{array}{cc} \frac{\sin\left(\pi x\right)}{\pi x}, & x\neq 0,\\ 1, & x=0. \end{array} \end{array}$$

If *f* is defined on ℝ, then for any *h* > 0 the series

(8) $$\begin{array}{c} C\left(f,h\right)\left(z\right)=\overset{\infty }{\underset{k=-\infty }{\sum }}f\left(kh\right)S\left(k,h\right)\left(z\right)‍ \end{array}$$

is called the Whittaker cardinal expansion of *f* whenever this series converges. *f* can be approximated by truncating (8).

To construct our needed approximations on the interval [*a*, *b*], we choose

(9) $$\begin{array}{c} \varphi \left(x\right)=\ln\left(\frac{x-a}{b-x}\right) \end{array}$$

which maps the finite interval [*a*, *b*] onto ℝ. The basic functions on [*a*, *b*] are taken to be the composite translated sinc functions:

(10) $$\begin{array}{c} S_{\varphi }\left(k,h\right)\left(x\right)=S\left(k,h\right)\left(\varphi \left(x\right)\right)=\text{sinc}\left(\frac{\varphi \left(x\right)-kh}{h}\right). \end{array}$$

Thus we may define the inverse image of the equidistant space node {*ih*} as

(11) $$\begin{array}{c} x_{i}=\varphi^{-1}\left(ih\right)=\frac{a+be^{ih}}{1+e^{ih}},\;i=0,\pm 1,\pm 2,\ldots . \end{array}$$

The class of functions such that the known exponential convergence rate exists for the sinc interpolation is denoted by *B*(*D*_*E*_) and defined in the following text.

**Definition 3 (see [21])**

Let *B*(*D*_*E*_) be the class of functions *f* which are analytic in *D*_*E*_ and satisfy

(12) $$\begin{array}{c} \int_{\varphi^{-1}(x+L)}\left|f\left(z\right)dz\right|‍⟶0,\;x⟶\pm \infty , \end{array}$$

where *L* = {*iυ* : |*υ* | <*d* ≤ *π*/2}, and

(13) $$\begin{array}{c} \int_{\partial D_{E}}\left|f\left(z\right)dz\right|‍<\infty \end{array}$$

on the boundary of *D*_*E*_ (denoted ∂*D*_*E*_).

**Theorem 4 (see [21, 23])**

If *φ*′*f* ∈ *B*(*D*_*E*_), then, for all *x* ∈ [*a*, *b*],

(14) $$\begin{array}{c} \left|f\left(x\right)-\overset{\infty }{\underset{k=-\infty }{\sum }}f\left(x_{k}\right)S_{\varphi }\left(k,h\right)\left(x\right)‍\right|\leq \frac{2N\left(\varphi^{\prime }f\right)}{\pi d}e^{-\pi d/h}. \end{array}$$

Further, one assumes that there are positive constants *C* and *β* so that |*f*(*x*)|≤*C*exp⁡(−*β* | *φ*(*x*)|). And if one selects $h=\sqrt{\pi d/\beta N}\leq 2\pi d/\ln2$, then,

(15) |dmf(x)dxm−∑k=−NNf(xk)dmdxmSφ(k,h)(x)‍|  ≤KN(m+1)/2exp⁡(−πdβN)

for all *m* = 0,1,…, *n*.

The above expressions show that the sinc interpolation on *B*(*D*_*E*_) converges exponentially. We also require the following derivatives of the composite translated sinc functions evaluated at the nodes. Consider

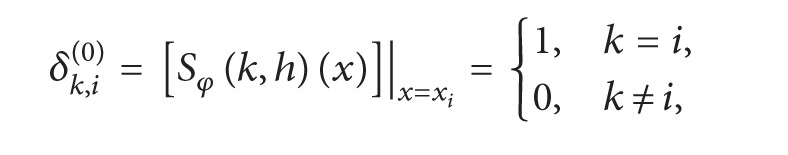
(16)

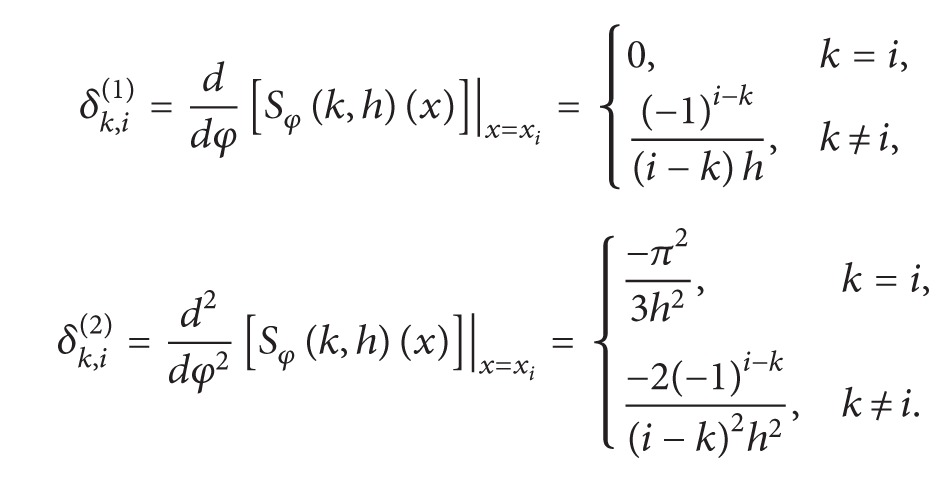
(17)


### 2.3. The Shifted Chebyshev Polynomials

The Chebyshev polynomials {*T*_*i*_(*σ*);  *i* = 0,1,…} are a well-known family of orthogonal polynomials defined on the interval [−1,1] and can be determined with the aid of the recurrence formulae [25, 26]:

(18) $$\begin{array}{c} T_{n+1}\left(\sigma \right)=2xT_{n}\left(\sigma \right)-T_{n-1}\left(\sigma \right),\;n=1,2,\ldots ,\\ T_{0}\left(\sigma \right)=1,\;T_{1}\left(\sigma \right)=\sigma . \end{array}$$

In order to use these polynomials on the interval *t* ∈ [0, *τ*], it is necessary to define the so-called shifted Chebyshev polynomials by the variable substitution: *σ* = (2*t*/*τ*) − 1. Let the shifted Chebyshev polynomials *T*_*i*_((2*t*/*τ*) − 1) be denoted by *T*_*τ*,*i*_(*t*). The analytic form of the shifted Chebyshev polynomials *T*_*τ*,*i*_(*t*) is given by

(19) $$\begin{array}{c} T_{\tau ,i}\left(t\right)=i\overset{i}{\underset{k=0}{\sum }}{\left(-1\right)}^{i-k}\frac{\left(i+k-1\right)!2^{2k}}{\left(i-k\right)!\left(2k\right)!\tau^{k}}t^{k}‍,\;i=1,2,\ldots ,\\ T_{\tau ,0}\left(t\right)=1. \end{array}$$

Specially, *T*_*τ*,*i*_(0) = (−1)^*i*^ and *T*_*τ*,*i*_(*τ*) = 1.

Caputo's fractional derivative of order *α* > 0 for the shifted Chebyshev polynomials *T*_*τ*,*i*_(*t*) is given by

(20) D0αCTτ,i(t)=∑k=⌈α⌉ibi,ktk−α‍, i=⌈α⌉,⌈α⌉+1,…,D0αCTτ,i(t)=0, i=0,1,…,⌈α⌉−1,

where

(21) $$\begin{array}{c} b_{i,k}=i{\left(-1\right)}^{i-k}\frac{\left(i+k-1\right)!2^{2k}k!}{\left(i-k\right)!\left(2k\right)!\tau^{k}\Gamma \left(k-\alpha +1\right)}. \end{array}$$

## 3. The Derivation of the Sinc-Chebyshev Collocation Method

In order to solve problem (1) with (2) and (3), first of all, we approximate *u*(*x*, *t*) by the 2*m* + 1 composite translated sinc functions and *n* + 1 shifted Chebyshev polynomials as

(22) $$\begin{array}{c} u_{m,n}\left(x,t\right)=\overset{m}{\underset{i=-m\;\;}{\sum }}\overset{n}{\underset{j=0}{\sum }}c_{ij}S_{\varphi }\left(i,h\right)\left(x\right)T_{\tau ,j}\left(t\right)‍‍. \end{array}$$

It is noted that the approximate solution *u*_*m*,*n*_(*x*, *t*) satisfies the boundary conditions in (3) since *S*_*φ*_(*i*, *h*)(*x*), *i* = −*m*, −*m* + 1,…, *m*, tend to zeros when *x* tends to *a* and *b*. For discretizing (1) with (2), the lemma is given as follows.

**Lemma 5**

Let 1 < *α* < 2 and *x*_*k*_ be spatial collocation points given in (11). Then the following relations hold:

(23) $$\begin{array}{c} \frac{\partial^{\alpha }u_{m,n}\left(x_{k},t\right)}{\partial t^{\alpha }}=\overset{n}{\underset{j=2\;\;}{\sum }}\overset{j}{\underset{r=2}{\sum }}c_{kj}b_{j,r}t^{r-\alpha }‍‍,\\ \frac{\partial^{2}u_{m,n}\left(x_{k},t\right)}{\partial x^{2}}=\overset{m}{\underset{i=-m\;\;}{\sum }}\overset{n}{\underset{j=0}{\sum }}c_{ij}q_{i,k}T_{\tau ,j}\left(t\right)‍‍,\\ \frac{\partial u_{m,n}\left(x_{k},t\right)}{\partial t}=\overset{n}{\underset{j=1\;\;}{\sum }}\overset{j}{\underset{r=1}{\sum }}c_{kj}d_{j,r}t^{r-1}‍‍, \end{array}$$

where *q*_*i*,*k*_ = *φ*′′(*x*_*k*_)*δ*_*i*,*k*_^(1)^ + [*φ*′(*x*_*k*_)]^2^ *δ*_*i*,*k*_^(2)^ and *d*_*j*,*r*_ = *j*(−1)^*j*−*r*^(*r*(*j* + *r* − 1)!2^2*r*^/(*j* − *r*)!(2*r*)!*τ* ^*r*^).

**Proof**

By (16), (20), and (22), it follows that

(24) ∂αum,n(xk,t)∂tα=∑i=−m  m‍∑j=0n‍cijSφ(i,h)(xk)D0αCTτ,j(t)=∑i=−m  m∑j=2ncijδi,k(0)∑r=2jbj,rtr−α‍‍‍=∑j=2  n∑r=2jckjbj,rtr−α‍‍.

Taking into account (17), we obtain

(25) ∂2um,n(xk,t)∂x2 =∑i=−m  m∑j=0ncijd2dx2[Sφ(i,h)(x)]|x=xkTτ,j(t)‍‍ =∑i=−m  m∑j=0ncij(φ′′(xk)δi,k(1)+[φ′(xk)]2δi,k(2))Tτ,j(t)‍‍ =∑i=−m  m∑j=0ncijqi,kTτ,j(t).‍‍

Using (16) and (19), one has

(26) ∂um,n(xk,t)∂t=∑i=−m  m∑j=0ncijSφ(i,h)(xk)ddtTτ,j(t)‍‍=∑i=−m  m∑j=1ncijδi,k(0)j∑r=1j(−1)j−rr(j+r−1)!22r(j−r)!(2r)!τrtr−1‍‍‍=∑j=1  n∑r=1jckjdj,rtr−1‍‍.

The proof is completed.

We are now ready to solve problem (1) with (2) and (3). A collocation scheme is constructed by substituting (22) for *u*(*x*, *t*) into (1) and evaluating the result at the points *x*_*k*_ in (11) and *t*_*l*_. For suitable temporal collocation points, we use the roots *t*_*l*_ (*l* = 1,2,…, *n* − 1) of the shifted Chebyshev polynomials *T*_*τ*,*n*−1_(*t*). Therefore, using Lemma 5, we have

(27) ∑j=2  n∑r=2jckjbj,rtlr−α‍‍ −a(xk,tl)∑i=−mm ∑j=0ncijqikTτ,j(tl)‍‍=f(xk,tl),k=−m,−m+1,…,m,  l=1,2,…,n−1.

Also by applying (22) to the initial conditions (2) and collocating in 2*m* + 1 points *x*_*k*_, we obtain

(28) $$\begin{array}{c} \overset{n}{\underset{j=0}{\sum }}{\left(-1\right)}^{j}c_{kj}=\phi \left(x_{k}\right)‍,\;k=-m,-m+1,\ldots ,m,\\ \overset{n}{\underset{j=1}{\sum }}{\left(-1\right)}^{j-1}\frac{2j^{2}}{\tau }c_{kj}=\psi \left(x_{k}\right)‍,\;k=-m,-m+1,\ldots ,m. \end{array}$$

To obtain a matrix representation of the above equations, we let

(29) A=[A1A2A3],  B=[B1B2B3],C=[c11,c12,…,c1,n+1,c21,c22,…,c2,n+1,…,c2m+1,1,c2m+1,2,…,c2m+1,n+1]T,p=⌊i(2m+1)⌋,  s=⌊j(n+1)⌋,v=j−(s−1)(n+1)−1,Δ=a(xi−p(2m+1)−m−1,tp)qs,i−p(2m+1)−m−1Tτ,v(tp),

where

(30) A1=(aij(1))(2m+1)×[(2m+1)(n+1)],A2=(aij(2))(2m+1)×[(2m+1)(n+1)],A3=(aij(3))[(2m+1)(n−1)]×[(2m+1)(n+1)],aij(1)={(−1)j−(i−1)(n+1)−1,1≤j−(i−1)(n+1)≤n+1,0,else,aij(2)={(−1)j−(i−1)(n+1)2[j−(i−1)(n+1)−1]2τ,2≤j−(i−1)(n+1)≤n+1,0,else,aij(3)={Δ+∑l=2vbv,ltpl−α‍,s=i−p(2m+1),  v≥2,Δ,  else,B1=[ϕ(x−m),ϕ(x−m+1),…,ϕ(xm)]T,B2=[ψ(x−m),ψ(x−m+1),…,ψ(xm)]T,B3=[f(x−m,t1),f(x−m+1,t1),…,f(xm,t1),f(x−m,t2),f(x−m+1,t2),…,f(xm,t2),…,f(x−m,tn−1),f(x−m+1,tn−1),…,f(xm,tn−1)]T.

So we get a system of (2*m* + 1)(2*n* + 1) linear equations with (2*m* + 1)(2*n* + 1) unknown parameters *c*_*ij*_,  *i* = 1,2,…, 2*m* + 1,  *j* = 1,2,…, *n* + 1. And this system can be expressed in a matrix form

(31) $$\begin{array}{c} AC=B. \end{array}$$

Equation (31) can be solved easily for the unknown coefficients *c*_*ij*_. Consequently *u*_*m*,*n*_(*x*, *t*) given in (22) can be calculated.

## 4. Numerical Examples

To validate the effectiveness of the proposed method for problem (1) with (2) and (3), we consider the example given in [16].

(32) $$\begin{array}{c} \frac{\partial^{\alpha }u\left(x,t\right)}{\partial t^{\alpha }}=\frac{\partial^{2}u\left(x,t\right)}{\partial x^{2}}+\sin\left(\pi x\right),\;0<x<1,\;\;0<t\leq 1,\\ u\left(x,0\right)=0,\;\frac{\partial u\left(x,0\right)}{\partial t}=0,\;0<x<1,\\ u\left(0,t\right)=0,\;\;u\left(1,t\right)=0,\;0<t\leq 1. \end{array}$$

The exact solution of the above problem is [27]

(33) $$\begin{array}{c} u\left(x,t\right)=\frac{1}{\pi^{2}}\left[1-E_{\alpha }\left(-\pi^{2}t^{\alpha }\right)\right]\sin\left(\pi x\right), \end{array}$$

where *E*_*α*_(*z*) = ∑_*k*=0_^*∞*^ *z* ^*k*^/Γ(*αk* + 1) is the one-parameter Mittag-Leffler function.

**Figure 1:**
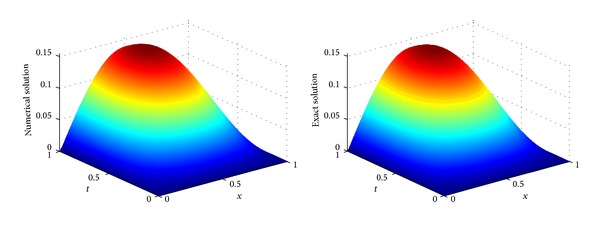
Comparison of the numerical and exact solution in the domain [0,1]×[0,1].

**Figure 2:**
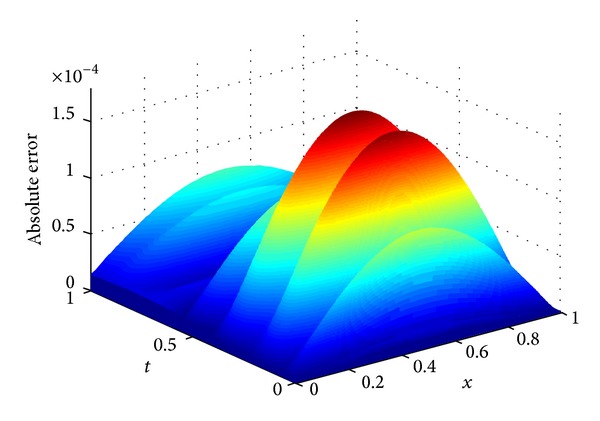
Plot of the absolute error.

**Figure 3:**
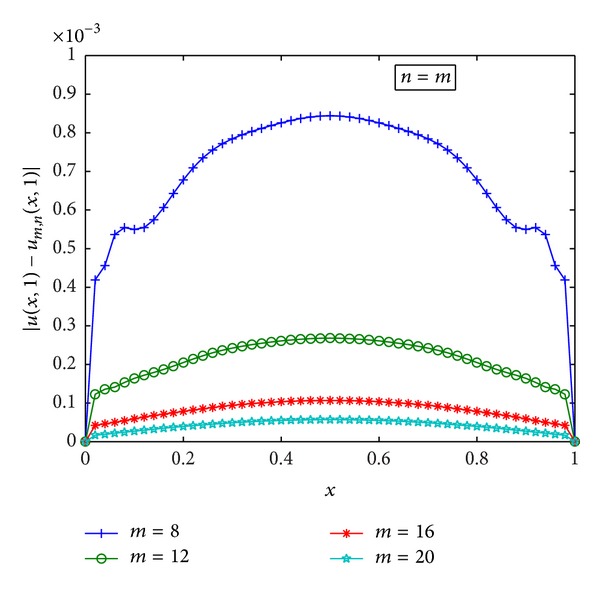
The error curves at *t* = 1.

To solve the above problem with *α* = 1.7 by using the method described in Section 3, we choose *β* = 1 and *d* = *π*/2, and this leads to $h=\pi /\sqrt{2m}$. We will report the accuracy and efficiency of the method based on the *L* ^2^-errors and *L* ^*∞*^-errors.  Figure 1 gives the 3D diagrams of the numerical and exact solutions on the whole computational domain [0,1]×[0,1] with *m* = 15,  *n* = 8. A good agreement of the numerical solution with the exact one is achieved. In Table 1, we list the numerical and exact solutions at some points for different numbers of collocation points with *n* = *m*. Furthermore, Figure 2 shows the absolute error function |*u*(*x*, *t*) − *u*_*m*,*n*_(*x*, *t*)| obtained by the presented method with *m* = 15 and *n* = 8. In Figure 3, we plot the curves of the absolute errors at *t* = 1 for different numbers of collocation points. From Figures 2 and 3, we see that the proposed method can provide accurate results only using a small number of collocation points.

**Figure 4:**
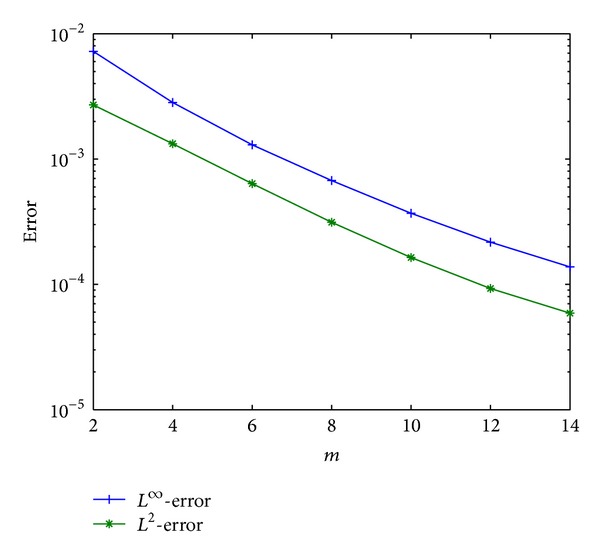
*L* ^2^- and *L* ^*∞*^-errors versus *m*.

To explore the dependence of errors on the parameters *m*, *n*, we represent the *L* ^*∞*^-error and *L* ^2^-error in semi-log scale. Firstly, the computational investigation is concerned with the spatial error. To this end, we fix the polynomial degree *n* = 20, a value large enough such that the error stemming from the temporal approximation is negligible. In Figure 4, we plot the error as functions of *m*, where a logarithmic scale is used for the spatial-error-axis. As expected, the error shows an exponential decay, since in this semi-log representation one observes that the error variations are approximately linear versus *m* [19].

**Figure 5:**
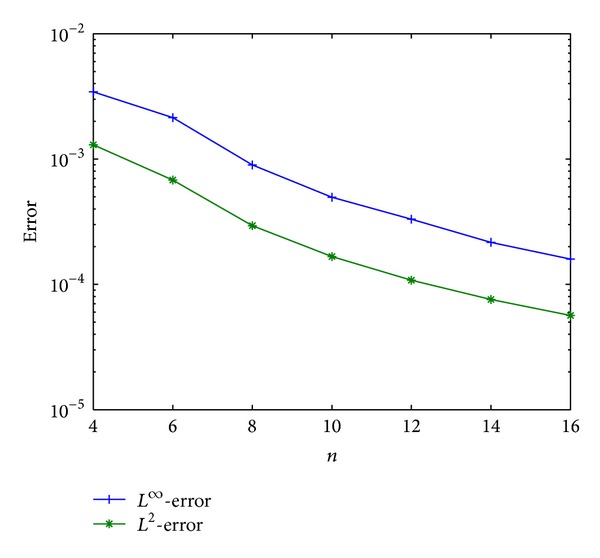
*L* ^2^- and *L* ^*∞*^-errors versus *n*.

Now we check the temporal error, which is more interesting because of the fractional derivative in time. For a similar reason mentioned above, we fix a large enough value *m* = 20 to avoid contamination of the spatial error. We present the error as a function of the shifted Chebyshev polynomial degree *n* in Figure 5, where a logarithmic scale is now used for the temporal-error-axis. From Figure 5, it is clearly observed that the temporal error depends on the discretization parameters *n*.

## 5. Conclusion

In this paper, we develop and analyze the efficient numerical methods for the fractional diffusion-wave equation. Based on the collocation technique, the sinc functions and shifted Chebyshev polynomials are used to reduce the problem to the solution of a system of linear algebraic equations. And a matrix representation of the above equations is obtained. In the numerical example, the solution obtained by this method is in excellent agreement with the exact one. The effectiveness and convergence of the presented method are confirmed through the numerical experimentation. One issue of future work is to develop the theory analysis of the method for the proposed fractional differential equation.

## Figures and Tables

**Table 1:** Some numerical and exact solutions at *t* = 1.

*m*∖(*x*, *t*)	(0.1,1)	(0.2,1)	(0.3,1)	(0.4,1)	(0.5,1)
8	0.042229	0.080693	0.111213	0.130835	0.137592
12	0.042615	0.081166	0.111755	0.131400	0.138168
16	0.042720	0.081293	0.111903	0.131557	0.138330
20	0.042752	0.081332	0.111948	0.131606	0.138379
Exact solution	0.042779	0.081371	0.111997	0.131661	0.138436
